# *Arbuscular mycorrhizal fungi* regulate the peanut rhizosphere microbiome to alleviate salinity stress and enhance yield

**DOI:** 10.3389/fmicb.2025.1739241

**Published:** 2026-03-18

**Authors:** Ying-chun Du, Di Wang, Yu-qi Song, Qing-song Zheng, Lin Wang

**Affiliations:** 1 College of Rural Revitalization, Jiangsu Open University, Nanjing, Jiangsu, China; 2 College of Resources and Environmental Science, Jiangsu Provincial Key Laboratory of Coastal Saline Soil Resources Utilization and Ecological Conservation, Nanjing Agricultural University, Nanjing, Jiangsu, China

**Keywords:** *arbuscular mycorrhizal* fungi, microbial resilience, peanut, rhizosphere microbiome, salt stress

## Abstract

Salt stress threatens peanut yield by impairing physiological performance and disrupting rhizosphere microbial community stability. To investigate how *arbuscular mycorrhizal fungi* (AMF) mediate plant–microbe interactions under salt stress, researchers conducted a controlled pot experiment with four treatments: non-salt control (CK), AMF inoculation (A), salt stress (S; 100 mM sodium chloride), and combined AMF inoculation under salt stress (SA), with five biological replicates in each group. Plant growth traits, yield, and rhizosphere bacterial community were assessed at different peanut growth stages. AMF inoculation significantly increased peanut biomass, chlorophyll content, and yield, both under salt and non-salt stress conditions. Salt stress significantly reduced bacterial richness and community evenness, while AMF partially restored *α*-diversity and reshaped bacterial community composition. Functional predictions indicated that AMF enriched nitrogen cycling pathways such as nitrate reduction and nitrogen fixation. Furthermore, AMF promoted a more complex and stable bacterial community under salt stress, characterized by enhanced synergistic effects among key taxa, including Actinobacteria, Firmicutes, and Proteobacteria. Overall, AMF inoculation enhanced plant performance and rhizosphere bacterial resistance, highlighting its potential as an effective ecological strategy for improving peanut yield in saline-alkali agricultural ecosystems.

## 1 Introduction

Soil salinization has become one of the most serious environmental problems limiting agricultural productivity and threatening global food security. According to the Food and Agriculture Organization (FAO) (Zhou et al., 2024) approximately 833 million hectares of land worldwide are affected by salinity, representing around 7% of the Earth’s total land area. In addition, salinity threatens more than 20% of the world’s irrigated agricultural land, where intensive cultivation and inadequate drainage accelerate salt accumulation (Liang et al., 2024). The accumulation of soluble salts, primarily sodium chloride (NaCl) and sodium sulfate (Na_2_SO_4_), alters soil structure, increases osmotic potential, and disrupts nutrient uptake and water balance in plants. In China, salinized land exceeds 36 million hectares, concentrated mainly in northern and coastal regions such as Shandong, Jiangsu, and Xinjiang, where peanut cultivation is common (Huang et al., 2025). Peanuts (*Arachis hypogaea L.*), being moderately salt-sensitive legumes, experience significant yield losses under saline-alkali conditions. Excess Na^+^ in the rhizosphere interferes with K^+^ and Ca^2+^ uptake, reduces photosynthetic efficiency, inhibits nodule formation, and disrupts nitrogen fixation (Sá et al., 2019). Physiological studies have shown that a NaCl concentration above 75–100 mM can reduce peanut germination rate, chlorophyll content, and dry matter accumulation by more than 40% (Li et al., 2025). Hence, enhancing peanut salt tolerance has become a critical research topic in sustainable oil crop production, especially for expanding cultivation into marginal saline lands.

AMF are ubiquitous soil microorganisms that form mutualistic associations with more than 80% of terrestrial plant species (Wu et al., 2013). Belonging to the phylum Glomeromycota, AMF colonize plant roots and develop extensive extraradical mycelium, facilitating nutrient and water uptake from the soil. Numerous studies have demonstrated that AMF improve plant tolerance to abiotic stresses, particularly salinity, drought, and heavy metal toxicity (Sensoy et al., 2013; Chowdhary and Songachan, 2025; Shirani Bidabadi and Mehralian, 2020; Cheng et al., 2021; Adeyemi et al., 2021). The mechanisms underlying AMF-mediated salt tolerance include osmotic adjustment, ion homeostasis, antioxidant defense, and enhanced nutrient acquisition (Zhang et al., 2023; Abdel Motaleb et al., 2020; Shi-Chu et al., 2019). Under saline conditions, AMF symbiosis increases K^+^ and Ca^2+^ uptake while reducing Na^+^ accumulation, thus maintaining a favorable K^+^/Na^+^ ratio critical for enzyme activation and photosynthetic function (Chen et al., 2022). For example, inoculation with Rhizophagus irregularis significantly improved maize growth and leaf water potential under 100 mM NaCl (Chen et al., 2022). Similar effects have been observed in wheat, tomato, rice, and soybean, indicating that AMF mitigate salt stress through both physiological and biochemical modulation (Chen et al., 2022; Hui et al., 2025; Pu et al., 2024). In peanuts, recent research has reported that AMF inoculation enhances root vigor, chlorophyll content, and nodule nitrogenase activity under saline stress (Pu et al., 2024; Ci et al., 2023). However, most previous studies focused on plant physiological responses, while the ecological mechanisms—particularly AMF’s influence on rhizosphere bacterial community dynamics under salinity—remain poorly understood (Sánchez-Roque et al., 2022).

The rhizosphere bacterial community plays a pivotal role in plant growth and stress adaptation by participating in nutrient cycling, hormone regulation, and disease suppression (Raaijmakers et al., 2009). Salt stress alters the composition and interaction networks of soil microbiota, often reducing microbial diversity and functional stability (Yuan et al., 2019). Beneficial microbial groups, including *Proteobacteria, Actinobacteria*, and *Bacteroidetes*, are often suppressed under high salinity, while halophilic or pathogenic taxa may become dominant (Liu et al., 2022; Guo et al., 2023). Recent advances in high-throughput sequencing and network analysis have revealed that microbial co-occurrence networks—representing potential ecological interactions among operational taxonomic units (OTUs)—can serve as indicators of ecosystem stability (Du et al., 2025). A more connected and modular microbial network often corresponds to higher resilience against environmental perturbations (Wang et al., 2025). Several studies have shown that AMF inoculation reshapes the rhizosphere microbial structure, promoting the enrichment of beneficial taxa and strengthening microbial connectivity (Wang et al., 2025; Ji et al., 2022; Wang et al., 2024). Despite these insights, how AMF-mediated microbial network reorganization contributes to peanut salt tolerance remains largely unexplored.

Understanding the interactions among AMF symbiosis, microbial network organization, and rhizosphere ecological functions is essential for developing sustainable strategies to enhance peanut tolerance in saline–alkali environments. Therefore, this study aims to investigate how inoculation with AMF influences peanut growth, yield, rhizosphere bacterial community structure, functional potential, and network stability under moderate salinity (100 mM NaCl). Specifically, we hypothesized that AMF inoculation would: (i) alleviate the inhibitory effects of salt stress on peanut growth and yield; (ii) restore or enhance rhizosphere microbial *α*-diversity and reshape community composition; and (iii) strengthen microbial co-occurrence network connectivity, modularity, and the abundance of keystone taxa, thereby improving the ecological stability and resilience of rhizosphere microbial communities. By integrating microbial ecology with plant physiology, this study provides new mechanistic insights into AMF–microbiome interactions and offers a theoretical basis for utilizing beneficial fungi to support peanut production in salt-affected soils.

## 2 Materials and methods

### 2.1 Experimental design

The experiment was conducted at the Jiangsu Hilly Region Agricultural Research Institute (Nanjing Institute of Agricultural Sciences, Jiangning District, Jiangsu Province, China; 31.717°N, 118.762°E). The study was conducted in a greenhouse with full daylight and a temperature maintained between 22 and 28 °C. Peanut cultivar “Taihua-8” was used. The peanut seeds were disinfected with 5% sodium hypochlorite and 70% alcohol, rinsed several times with sterile water, germinated in sterilized vermiculite for 3–4 days, and kept in the dark at 28 °C (Du et al., 2022). During germination, seedlings were directly inoculated with a spore suspension of *Rhizophagus irregularis* to ensure early symbiosis. When roots reached 2–3 cm in length, seedlings were transplanted into pots (22 cm diameter, 19 cm height, 10 kg soil) filled with normal soil. Four treatments were established: C-uninoculated control under non-saline conditions; A-inoculated with *R. irregularis* under non-saline conditions; S-100 mM NaCl stress without AMF inoculation; and SA-100 mM NaCl stress with *R. irregularis* inoculation. Each treatment was replicated three times, with five plants per replicate. After transplanting, AMF were allowed to establish for 7 days before salinization. Salt was applied by watering the plants with a 100 mM NaCl solution; a control group was irrigated with unsalted water, maintaining soil moisture at approximately 70% of field capacity. The original soil properties used were pH 7.03, electrical conductivity (EC) 159.47 μS/cm, total nitrogen (TN) 0.70 mg/g, available nitrogen (AN) 66.81 mg/kg, soil organic matter (SOM) 20 g/kg, available potassium (AK) 107.7 g/kg, and available phosphorus (AP) 20.8 mg/kg.

### 2.2 Growth and yield measurements

Twenty days after salt stress initiation, peanut plants from each treatment (CK, CK + AMF, S, and S + AMF) were randomly selected for growth assessment. Morphological and physiological parameters, including plant height, shoot and root fresh weights, and chlorophyll content (SPAD value), were recorded at this stage to evaluate early growth responses to AMF inoculation and salinity stress. Chlorophyll content was measured using a SPAD-502 Plus chlorophyll meter (Konica Minolta, Japan). At plant maturity, yield-related traits were determined for each treatment. The number of pods per plant, 100-pod weight, and yield per plant were measured as indicators of reproductive performance. Plants were manually harvested, and pods were oven-dried at 70 °C to constant weight before weighing. All measurements were conducted with three biological replicates per treatment. The data were expressed as the mean ± standard deviation (SD), and statistical significance among treatments was analyzed by one-way ANOVA followed by Tukey’s multiple range test (*p* < 0.05).

### 2.3 AMF colonization assessment

Peanut roots were collected, rinsed, and cut to ~1 cm. Segments were cleared in 10% KOH at 90 °C, rinsed, acidified in 1% HCl, and stained in 0.05% trypan blue (lactoglycerol) at 90 °C. Samples were destained overnight in lactoglycerol and examined under light microscopy to record hyphae, arbuscules, and vesicles (Phillips and Hayman, 1970). AMF colonization was quantified by the gridline-intersect method on 30–50 segments per replicate. Colonization (%) = positive intersections/total × 100. Across AMF-inoculated treatments, colonization typically ranged from approximately 40 to 80%, confirming successful establishment of the symbiosis, whereas non-inoculated treatments showed no detectable colonization. Data are mean ± SE, *n* = 3 biological replicates (five plants pooled). Treatments lacking AMF structures were reported as ND; differences were tested by one-way ANOVA (Tukey, *p* < 0.05). Safety precautions were observed.

### 2.4 Sample collection, DNA extraction

Rhizosphere soil samples were collected from potted peanut plants at three key growth stages: seedling stage (S), flowering stage (F), and maturity stage (M). Each pot was considered one biological replicate, and three replicates were sampled per treatment at each growth stage. In total, 36 rhizosphere soil samples were obtained (4 treatments × 3 growth stages × 3 replicates). Rhizosphere soil was carefully collected by gently shaking off the loosely attached soil from the roots, followed by brushing and collecting the tightly adhering soil surrounding the root surface. The obtained soils were homogenized and passed through a 2-mm sieve to remove visible plant residues and stones. Each soil sample was then divided into two portions: one portion was immediately frozen in liquid nitrogen and stored at −80 °C for DNA extraction, while the remaining portion was air-dried for backup.

### 2.5 MiSeq sequencing and sequence data processing

The V3-V4 hypervariable regions of the bacterial 16S rRNA gene were amplified from the soil genomic DNA using the primer 341F (5′-CCTACGGGNGGCWGCAG-3′) and 806R (5′-GGACTACHVGGGTWTCTAAT3′). The primer pair 341F/806R was selected because it targets the V3–V4 hypervariable regions of the bacterial 16S rRNA gene, which provide a favorable balance between taxonomic resolution and sequencing accuracy. This primer set has been widely used and validated in soil and rhizosphere microbiome studies, demonstrating broad bacterial coverage and minimal amplification bias across diverse taxa. In addition, the V3–V4 region is well represented in the SILVA (138.2) reference database, allowing reliable taxonomic assignment of amplicon sequence variants (ASVs). The PCR conditions consisted of an initial denaturation at 98 °C for 30 s, followed by 30 cycles of denaturation at 98 °C for 10 s, annealing at 55 °C for 30 s, and extension at 72 °C for 30 s, with a final extension at 72 °C for 5 min. All reactions were conducted in a Bio-Rad T100 thermal cycler. Amplicons were visualized on 2% agarose gels, purified using the Agencourt AMPure XP system (Beckman Coulter, USA), and subsequently used for library construction. The libraries were sequenced on an Illumina MiSeq platform at Lingen Bio-Pharm Technology Co. Ltd. (Shanghai, China). Amplicon sequencing bioinformatics was performed with EasyAmplicon v1.0. The paired-end sequence data were merged, quality filtered and de-replicated using VSEARCH v2.15 (Rognes et al., 2016). Then, the non-redundant sequences were de-noised into amplicon sequence variants (ASVs) with USEARCH v10.0 (Edgar, 2010), enabling single-nucleotide resolution without similarity-based clustering. Accordingly, no OTU clustering threshold (e.g., 97% or 99% sequence similarity) was applied. Chimaeras were removed by applying VSEARCH against the SILVA database (Quast et al., 2013). The taxonomy of the features (ASVs) was classified by the USEARCH sintax algorithm in RDP training set 16 (Cole et al., 2014). All raw sequence data have been made available in the National Genomics Data Center (NGDC) database (GSA) under the access number CRA032385.

### 2.6 *α*- and *β*-diversity analysis

Analysis of the *α*-diversity (the numbers and abundances of taxa within communities) and *β*-diversity (compositional dissimilarity between communities) metrics were carried out using the vegan package, and the diversities were visualized by using the ggplot2 package in R v4.0.2. Principal coordinate analysis (PCoA) was performed on the distance matrices. Analysis of variance (PERMANOVA, 999 permutations) was performed to evaluate the significant differences among the microbial compositions of the samples. For the separate in-depth analyses of each sample type, we applied a sequence count threshold to the ASV tables. Specifically, we retained ASVs with at least two sequences (to remove singletons) that were detected in at least six samples (corresponding to the number of replicates per treatment). ASVs remaining after this filtering step were considered to represent the soil microbial communities. The resulting ASV tables were normalized using the trimmed mean of M-values (TMM) method, and the normalized values were expressed as relative abundance counts per million (CPM).

### 2.7 Identification of sensitive ASVs and co-occurrence networks

Sensitive ASVs were identified according to the method of Hartman et al. (2018). Briefly, we conducted indicator species analysis with the R package indicspecies (De Cáceres et al., 2010) to calculate the correlation coefficient (r) of each ASV’s positive association with rice cropping patterns. The analysis was conducted with 9,999 permutations and was considered significant at *p* < 0.05. We then tested for differential ASV abundances between one or more of the growth stages of the two cropping communities using likelihood ratio tests with the R package edgeR (Robinson et al., 2010) ASVs whose abundances were identified as differing among growth stages of the two cropping patterns at a false discovery rate (FDR)-corrected value of *p* < 0.05 were considered to be cropping pattern-responsive. Finally, we defined ASVs that were confirmed by both the indicator species analysis and likelihood ratio tests as cropping-sensitive ASVs (sensitive ASVs). Furthermore, we constructed two types of networks: one visualized the significant (*p* < 0.05) ASV associations with different cropping patterns from the indicator species analysis using bipartite networks, and the other utilized the TMM-normalized CPM counts to calculate Spearman rank correlations between ASVs and visualized the positive, significant correlations (*r* > 0.8 and *p* < 0.01). In the second cooccurrence network, we calculated the network properties, including the total number of network nodes (representing ASVs), the total number of edges (connections between nodes representing positive, significant correlations between ASVs), and the degrees of co-occurrence (number of direct correlations to a node); additionally, we identified the network modules, which are substructures of nodes with higher edge densities within groups than between groups, with the greedy optimization of modularity algorithm implemented in the R package igraph. All networks were visualized with the Fruchterman–Reingold layout with 9,999 permutations and were implemented in igraph (Clauset et al., 2004). Co-occurrence networks were constructed based on significant Spearman correlations to explore differences in microbial association patterns among treatments. Network significance was not evaluated against null or randomized models, as the analysis focused on comparative network structure rather than statistical deviation from random expectations.

### 2.8 Functional prediction of microbial communities

Functional annotations of prokaryotic taxa were carried out using FAPROTAX v.1.1 (Louca et al., 2016). The data was analyzed using R-studio. When an analysis consisted of only a control and an experimental group, an independent *t*-test was performed. When three or more groups were compared, one-way ANOVA was performed followed by a Duncan’s Test. The data were considered significantly different at *p* < 0.05.

## 3 Results

### 3.1 Morphological and growth responses of peanut under different treatments

Successful colonization of peanut roots by AMF was confirmed in AMF-inoculated treatments (Supplementary Figure S1). Typical mycorrhizal structures, including intraradical hyphae and vesicles, were observed in roots from the AMF (A) and AMF + salt (SA) treatments, whereas no colonization was detected in non-inoculated control (CK) or salt-stressed (S) plants (Supplementary Figure S1A). Quantitative analysis showed high colonization rates, exceeding 85% under non-saline conditions and remaining above 65% under salt stress (Supplementary Figure S1B), indicating effective AMF establishment even in saline soil.

**Figure 1:**
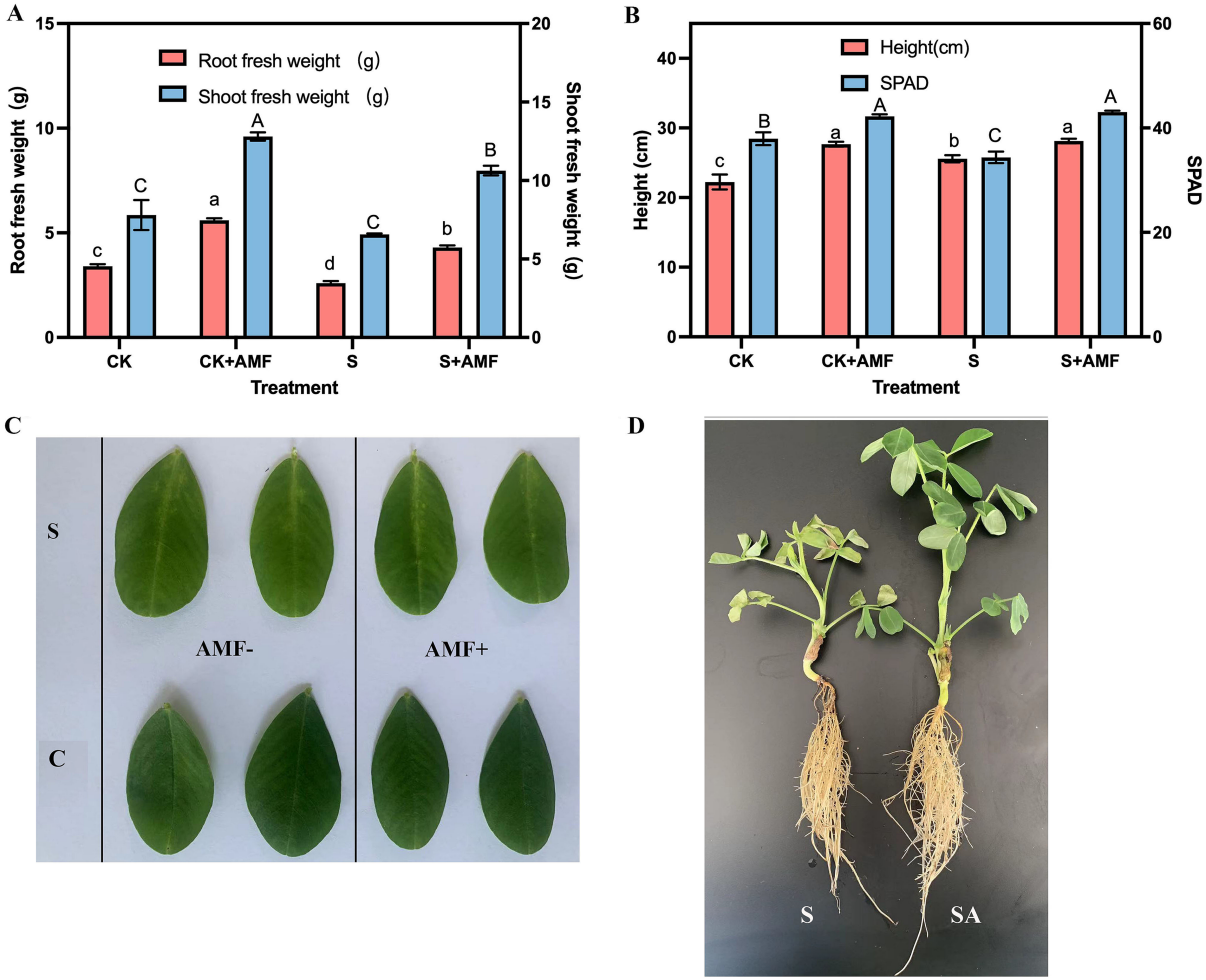
Effects of AMF inoculation on peanut growth and phenotype under salt stress. **(A)** Root and shoot fresh weights under different treatments. **(B)** Plant height and SPAD (chlorophyll content) under different treatments. **(C)** Phenotypic differences in peanut leaves showing that AMF inoculation alleviated leaf chlorosis under salt stress. **(D)** Representative images of peanut seedlings under salt stress (S) with or without AMF inoculation (S/SA). Different letters indicate significant differences among treatments (*p* < 0.05). Stages: S = seedling stage; F = flowering stage; M = maturity stage. Treatments: C = control; A = AMF inoculation; S = salt stress; SA = AMF inoculation under salt stress.

Following AMF colonization, significant differences in peanut growth and yield were observed among treatments (Figure 1). AMF inoculation increased root fresh weight by approximately 31% under non-saline conditions and 46% under salt stress, while shoot fresh weight increased by 43 and 61%, respectively (Figure 1A). Plant height and SPAD values were significantly higher in AMF-inoculated plants than in uninoculated controls (*p* < 0.05) (Figure 1B), and under salt stress, SPAD values in the S + AMF treatment were restored to levels comparable to CK. Morphological observations further supported these responses, as AMF-inoculated plants maintained greener leaves and more developed root systems under salinity (Figures 1C,D).

Yield-related traits showed similar trends (Table 1). Salt stress significantly reduced 100-pod weight and yield per plant, whereas AMF inoculation partially restored both parameters. The S + AMF treatment produced significantly higher yield and 100-pod weight than the S treatment alone, and CK + AMF also outperformed CK under non-saline conditions (*p* < 0.05). Overall, these results demonstrate that AMF colonization substantially alleviated the negative effects of salinity on peanut growth and productivity.

**Table 1:** Effects of AMF inoculation and salt stress on peanut yield and yield components.

Treatment	CK	A	S	SA
100 pods weight (g)	212.5 ± 1.1^a,b^	219.3 ± 1.2^a^	188.2 ± 1.4^c^	208.7 ± 0.9^b^
Yield (g/plant)	81.0 ± 0.6^b^	93.2 ± 0.8^a^	67.9 ± 0.5^c^	81.8 ± 0.6^b^
The number of total pods	14.1 ± 0.2^b^	15.0 ± 0.1^a^	12.2 ± 0.5^c^	13.7 ± 0.3^b^

Data represent the mean ± SD (*n* = 3). Different lowercase letters indicate significant differences among treatments within the same parameter (*p* < 0.05). Treatments: CK = control without AMF; A = AMF inoculation under non-saline conditions; S = salt stress; SA = AMF inoculation under salt stress.

### 3.2 Shifts in soil microbial diversity and functional composition

**Figure 2:**
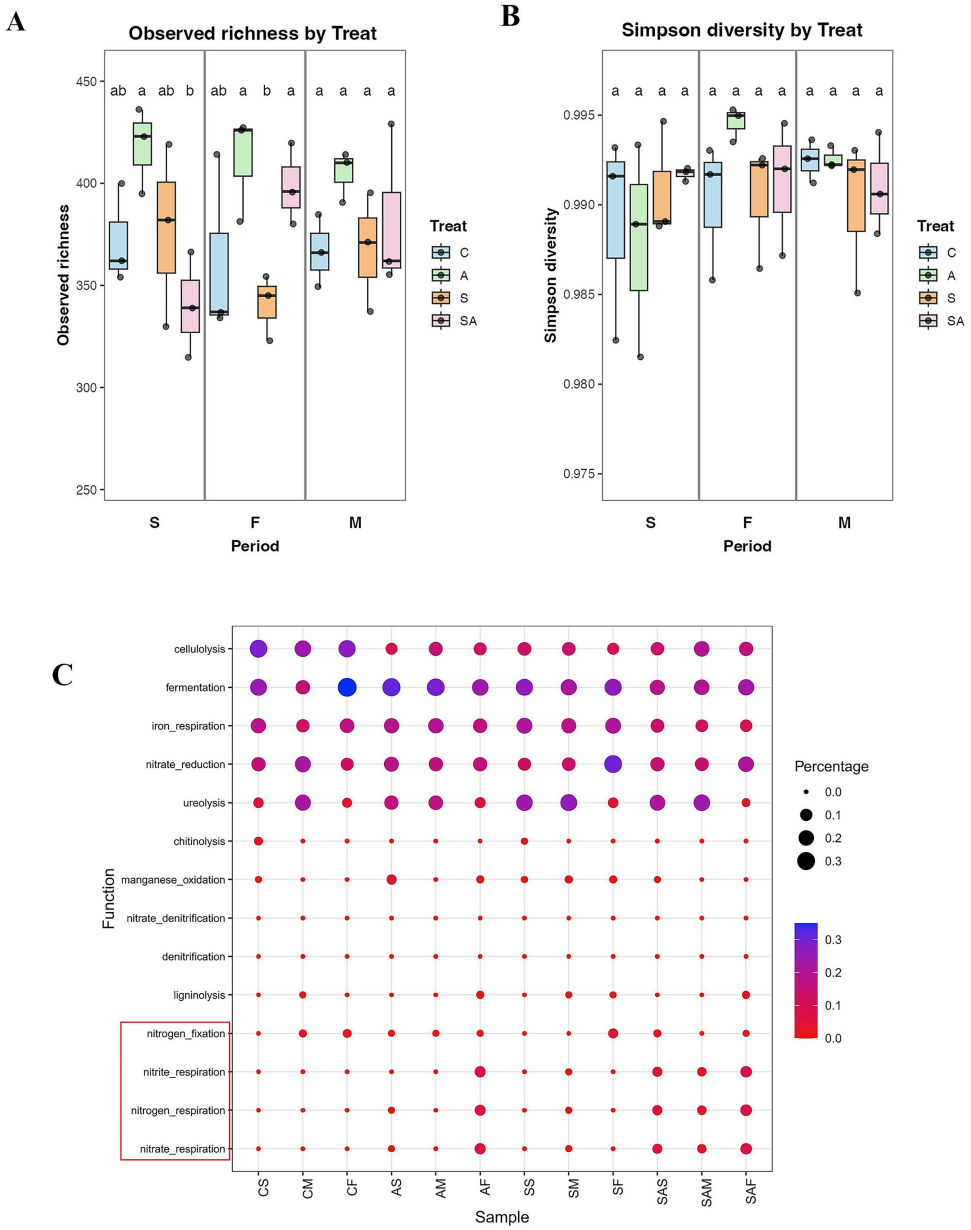
Alpha diversity and predicted microbial functions of rhizosphere bacteria under different treatments and growth stages. **(A)** Observed bacterial richness (Chao1 index). **(B)** Simpson diversity index. **(C)** Functional prediction of bacterial communities based on FAPROTAX annotation. The red box highlights nitrogen-related functions. Data represent means ± SD (*n* = 3). Different letters indicate significant differences among treatments within the same growth stage (*p* < 0.05). Stages: S = seedling stage; F = flowering stage; M = maturity stage. Treatments: C = control; A = AMF inoculation; S = salt stress; SA = AMF inoculation under salt stress.

Across the flowering and maturity stages, *α*-diversity metrics showed significant treatment effects (Figure 2A). At the flowering stage, bacterial richness (Chao1) was significantly reduced under salt stress compared with the control (*p* < 0.05), whereas AMF inoculation (A and SA) partially restored richness to levels not significantly different from CK (*p* > 0.05). Simpson diversity (Figure 2B) also decreased under salt stress (*p* < 0.05), but AMF inoculation mitigated this decline, with SA exhibiting significantly higher evenness than S (*p* < 0.05). By the maturity stage, richness and evenness recovered across treatments, although AMF-treated soils maintained slightly higher values, suggesting a sustained stabilizing effect of AMF on community structure.

Functional prediction based on the FAPROTAX database (Figure 2C) further demonstrated that AMF inoculation not only influenced community structure but also enhanced rhizosphere functional potential. Across both stages, AMF—associated soils exhibited higher proportions of nitrogen-related processes-including nitrogen fixation, nitrate respiration, nitrite respiration, and nitrogen respiration—compared with non-inoculated or salt-stressed treatments. In contrast, salt stress alone led to a decline in these functions and favored metabolic pathways related to fermentation and ureolysis, indicating a shift toward reduced nitrogen cycling activity. The combined AMF + salt treatment (SA) partially restored nitrogen-cycling functions, suggesting that AMF buffers the loss of microbial function under salt stress by potentially maintaining a beneficial nitrogen-transforming bacterial community.

### 3.3 Impact of different treatments on soil microbial communities

**Figure 3:**
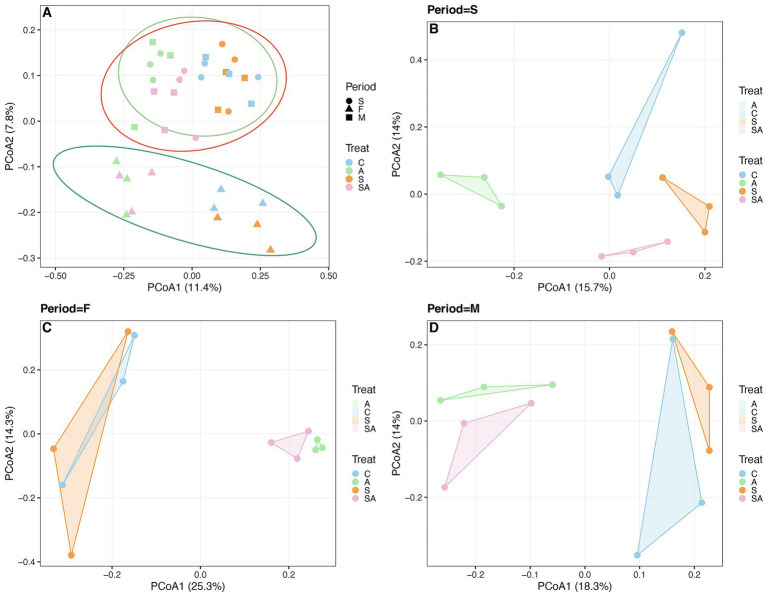
Principal coordinate analysis (PCoA) of bacterial community structure under different treatments across three growth stages. **(A)** Overall ordination of bacterial communities. **(B–D)** Separate PCoA plots for seedling (S), flowering (F), and maturity (M) stages. The percentages on the axes indicate the explained variation. Different colors and shapes represent treatments and growth stages, respectively. Stages: S = seedling stage; F = flowering stage; M = maturity stage. Treatments: C = control; A = AMF inoculation; S = salt stress; SA = AMF inoculation under salt stress.

Principal coordinate analysis (PCoA) based on Bray–Curtis distances showed that rhizosphere bacterial community composition differed among treatments and growth stages (Figure 3). The first two axes explained 11.4% (PCoA1) and 7.8% (PCoA2) of the total variation (Figure 3A). Despite the relatively low explained variance, samples exhibited consistent separation according to treatment and developmental stage, indicating that both AMF inoculation and salinity influenced bacterial community structure. At the seedling stage, clear separation among the four treatments (CK, A, S, and SA) was observed (Figure 3B). AMF-inoculated samples (A) were distinct from the control, while salt-stressed samples (S) (Figure 3B) formed a separate cluster. The combined treatment (SA) occupied an intermediate position between A and S. During the flowering stage, treatment-specific clustering persisted, although the distance between A and SA decreased (Figure 3C). By the maturity stage, overall clustering among treatments became less pronounced, but AMF-inoculated treatments (A and SA) remained distinct from non-inoculated treatments (CK and S) (Figure 3D).

To further assess stage-related variation within treatments, constrained analysis of principal coordinates (CAP) was performed with growth stage as the constraining factor (Supplementary Figure S2). In all treatments, bacterial community composition differed among growth stages, confirming that plant development was an important driver of community variation. However, the magnitude of stage-related separation varied among treatments: salt-stressed soils showed greater dispersion across stages, whereas AMF-inoculated treatments displayed more compact and consistent stage-associated patterns.

Together, the PCoA and CAP analyses indicate that rhizosphere bacterial communities were jointly shaped by treatment and plant developmental stage, and that AMF inoculation modified both overall community composition and stage-dependent variation under salt stress.

### 3.4 Identification of sensitive bacterial community (ASVs)

**Figure 4:**
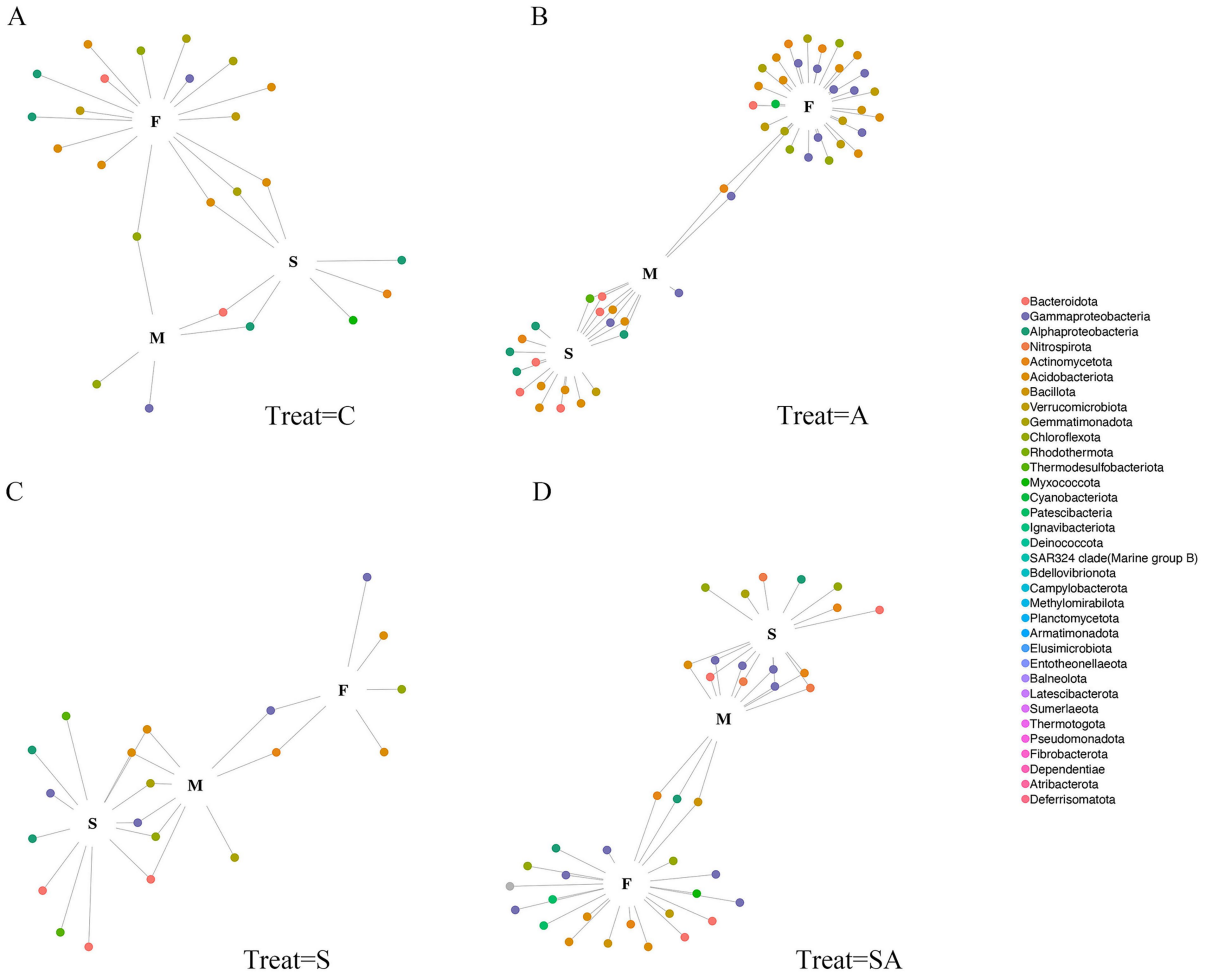
Identification of cultivation-sensitive ASVs across different treatments. Bipartite network diagrams show stage-specific indicator ASVs identified by indicator species analysis under different treatments: **(A)** Control (C), **(B)** AMF treatment (A), **(C)** Salt stress (S), and **(D)** Salt stress combined with AMF (SA). Large nodes represent peanut growth stages, while small colored nodes represent individual bacterial ASVs. Edges indicate ASVs that were positively and significantly associated with a specific growth stage (*p* < 0.05). ASVs are colored according to their taxonomic affiliations at the phylum level. Stages: S = seedling stage; F = flowering stage; M = maturity stage. Treatments: C = control; A = AMF inoculation; S = salt stress; SA = AMF inoculation under salt stress.

To determine the microbial taxa most responsive to different treatments, we performed indicator species analysis and visualized the results through bipartite network diagrams (Figure 4, Supplementary Table S1) and Venn plots (Supplementary Figure S3). The analysis revealed that bacterial ASVs were tightly clustered during the flowering and maturity stages, indicating that the composition of bacterial communities was shaped by plant developmental period. Across treatments, a substantial number of ASVs were shared between the two post-cultivation periods, reflecting consistent bacterial responses to plant growth. However, the distribution of indicator ASVs varied markedly among treatments. Specifically, the A and SA groups contained a greater number of treatment-specific nodes and stronger network connectivity compared with the S and C groups, implying that AMF inoculation enhanced bacterial responsiveness and interaction strength under both normal and saline conditions.

To ensure robustness, the identified indicator ASVs were further validated using likelihood-ratio tests in EdgeR. Only ASVs supported by both methods were defined as cultivation-sensitive ASVs (csASVs) (Supplementary Figure S3). In total, two csASVs were detected in the control (C) treatment, 14 in A, 4 in S, and 20 in SA. These findings indicate that AMF inoculation produced the largest shift in bacterial community composition. When comparing overlaps among treatments, less than one-third of the csASVs were shared (Supplementary Figure S3), suggesting that each treatment recruited a distinct subset of bacterial taxa. Although these ASVs responded to specific growth stages, they did not follow a consistent taxonomic pattern across stages (Supplementary Figures S4, S5). Overall, each growth phase fostered unique bacterial assemblages under different treatments, while a stable core microbiome persisted across treatments and periods, reflecting both treatment-specific and developmental-stage-driven microbial differentiation.

### 3.5 Impact of different treatments on microbial symbiotic patterns

**Figure 5:**
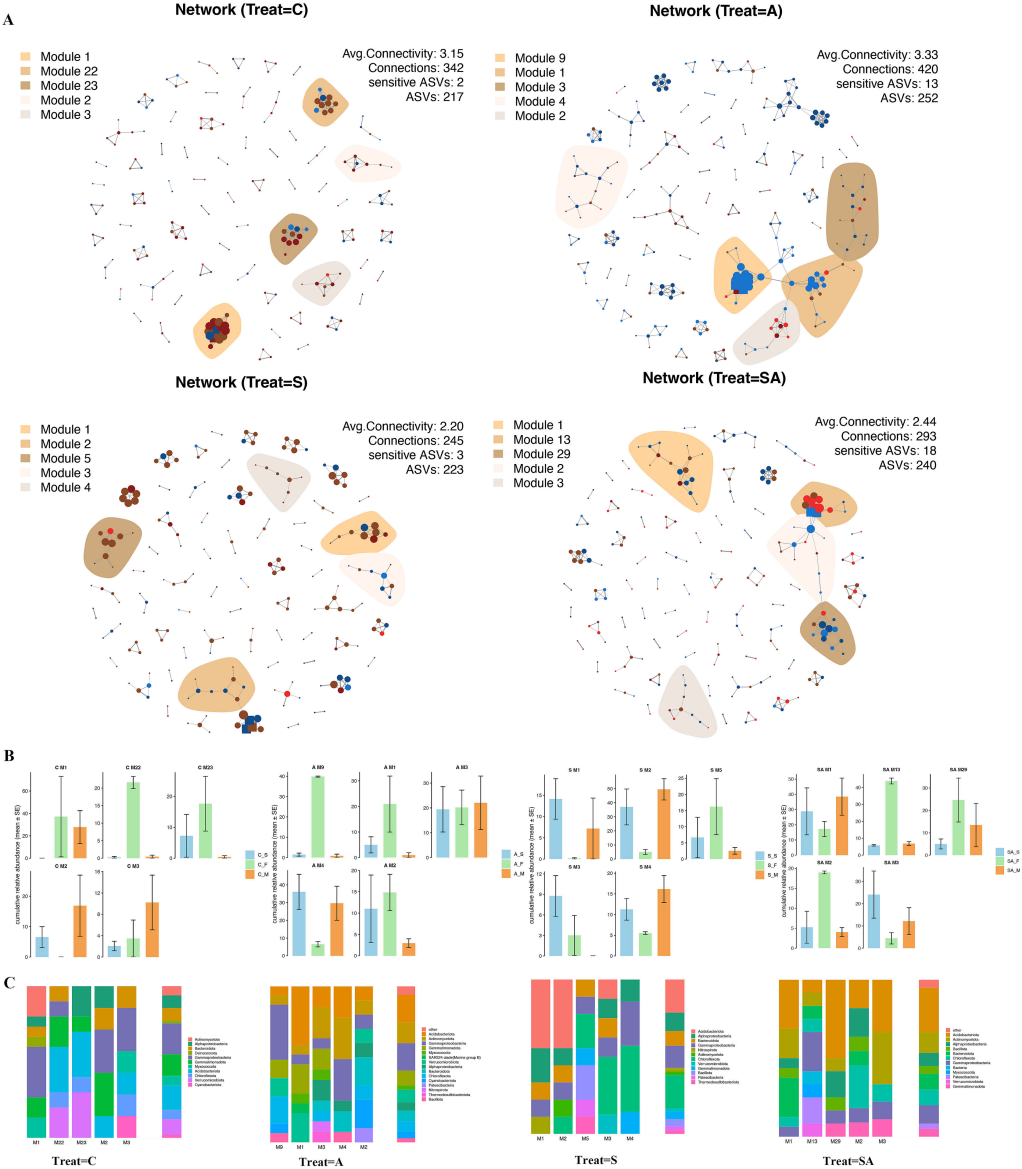
Co-occurrence patterns of cultivation sensitive ASVs. **(A)** Co-occurrence networks visualizing significant correlations (*p* > 0.7, *p* < 0.001; indicated with gray lines) between bacteria ASVs in soil communities. Circles indicate bacteria, and keystone ASVs are represented with asterisks (Table 1). ASVs are colored by their association to the different treatment. Shaded areas represent the network modules containing csASVs of all bacteria of the treatment sensitive modules in soil networks. The cumulative relative abundance in samples of different treatment indicates the overall response of treatment sensitive modules to the different stages. **(B)** Cumulative relative abundances (as counts per million, CPM; *y*-axis in ×1,000) of the modules in the different treatment networks. **(C)** Qualitative taxonomic composition of cultivation sensitive modules is reported as proportional ASVs numbers per class (bacteria) and compared to the overall taxonomic distribution in the entire dataset (column “all”) stages. ASVs are colored according to their phylum assignments. Stages: S = seedling stage; F = flowering stage; M = maturity stage. Treatments: C = control; A = AMF inoculation; S = salt stress; SA = AMF inoculation under salt stress.

Co-occurrence network analysis revealed clear differences in the organization of rhizosphere bacterial communities among treatments (Figure 5). Under the control treatment (C), the network comprised 217 ASVs and 342 edges, with an average connectivity of 3.15 (Figure 5A). Modules were mainly composed of Actinobacteria, Proteobacteria, and Firmicutes, reflecting a relatively balanced community structure. AMF inoculation (A) produced the most complex network, with 252 ASVs and 420 connections and the highest average connectivity (3.33). Several large and well-defined modules (e.g., M1, M2, M3, and M9) were observed, indicating enhanced bacterial co-occurrence and pronounced modular organization compared with the control. In contrast, the salt-stressed treatment (S) showed a simpler network structure, with fewer connections (245 edges) and the lowest average connectivity (2.20), suggesting constrained bacterial co-occurrence under salinity. Cultivation-sensitive ASVs in this treatment were mainly affiliated with Bacteroidetes, Alphaproteobacteria, and Deinococcota, but formed relatively sparse modules. The combined AMF + salt treatment (SA) displayed an intermediate network structure (240 ASVs, 293 connections; average connectivity 2.44). Compared with salt stress alone, the SA network showed increased connectivity and clearer modular patterns, although it did not reach the complexity observed under AMF inoculation alone, indicating partial mitigation of salinity-induced network simplification.

Figure 5B shows that the cumulative relative abundance of cultivation-sensitive modules varied among treatments and growth stages, reflecting differences in the abundance dynamics of treatment-responsive bacterial assemblages. Figure 5C further demonstrates that the taxonomic composition of these modules differed among treatments, indicating that distinct bacterial groups responded preferentially to AMF inoculation or salt stress.

## 4 Discussion

### 4.1 AMF alleviated salt stress and promoted peanut growth

In this study, AMF inoculation markedly improved peanut growth performance and yield under both normal and saline conditions. The enhanced biomass, plant height, and SPAD values indicate that AMF effectively mitigated salinity-induced inhibition of photosynthesis and nutrient uptake. These results are consistent with recent findings showing that AMF enhance host plant salt tolerance through improved ion homeostasis, osmotic adjustment, and antioxidant defense mechanisms (Chandrasekaran et al., 2014; Pellegrino et al., 2015). The denser and more extensive root system in AMF-inoculated plants also aligns with reports that mycorrhizal symbiosis facilitates water and nutrient acquisition under osmotic stress (Yang et al., 2015; Chandrasekaran, 2020).

Furthermore, AMF inoculation significantly increased pod weight and yield, especially under saline conditions. Similar improvements in reproductive success have been reported in other crops such as soybean and maize, where AMF enhanced carbon partitioning and nitrogen assimilation under stress (Suri et al., 2013; Barbosa et al., 2022). Collectively, these results suggest that AMF inoculation has practical potential as a biofertilizer-based strategy to support plant growth and productivity in salt-affected soils.

### 4.2 AMF modulated soil microbial diversity and functional potential

Soil microbial diversity serves as a key ecological bridge between AMF colonization and plant performance (Ma et al., 2022). Our alpha-diversity results revealed that salt stress suppressed microbial richness and evenness, while AMF inoculation reversed this trend, especially during the flowering and maturity stages. This observation supports the concept that AMF indirectly regulate rhizosphere microbiota through improved root exudation and nutrient availability (Gao et al., 2012; Aini et al., 2019). Although the first two PCoA axes explained a relatively limited proportion of the total variance (<20%), this pattern is common in complex soil bacterial community datasets. Therefore, PCoA was used primarily to visualize overall compositional trends, and the interpretation was supported by complementary analyses, including PERMANOVA and CAP, which confirmed significant effects of treatment and growth stage on bacterial community composition.

Functional prediction based on the FAPROTAX database showed enrichment of nitrogen-related processes—including nitrogen fixation, nitrate reduction, and nitrite respiration–under AMF inoculation. These processes contribute to improved soil fertility and nutrient turnover, consistent with recent evidence that AMF enhance nitrogen metabolism and microbial enzyme activities in saline–alkaline soils (dos Santos et al., 2017; Wang et al., 2019). In contrast, salt-stressed soils exhibited strong functional suppression, confirming that AMF play a restorative role in sustaining microbial nutrient cycling functions disrupted by salinity.

It should be noted that functional profiles inferred from FAPROTAX represent predicted metabolic potential based on taxonomic assignment rather than direct measurements of microbial activity. Therefore, the nitrogen-related functions highlighted here should be interpreted cautiously, and future studies incorporating metagenomics or metatranscriptomics are needed to confirm active nitrogen cycling processes.

### 4.3 Treatment-specific assembly and sensitivity of microbial taxa

Indicator species analysis and EdgeR validation revealed that AMF-treated soils (A and SA) contained a greater number of cultivation-sensitive amplicon sequence variants (csASVs), indicating that AMF inoculation was associated with stronger treatment-specific shifts in bacterial community composition. Similar treatment-specific microbial reassembly has been observed in AMF–crop systems, where inoculation modified root exudate profiles and consequently recruited unique bacterial partners (Du et al., 2024; Zhang et al., 2021).

In contrast, salt-stressed soils without AMF inoculation (S) exhibited fewer csASVs, suggesting a reduced magnitude of treatment-responsive bacterial taxa under salinity stress. This pattern reflects a constrained community response rather than enhanced adaptability. However, the persistence of a “core microbiome” across treatments implies functional redundancy and resilience, as reported in other AMF-associated systems (Liu et al., 2025). These findings collectively suggest that AMF inoculation does not merely restore pre-stress communities but drives distinct assembly trajectories that enhance rhizosphere stability and nutrient cycling.

It should be noted that the identification of cultivation-sensitive ASVs was based on a conservative framework combining indicator species analysis and edgeR testing. While this stringent filtering may underestimate the total number of responsive taxa, it increases confidence in the ecological relevance of the detected ASVs and reduces the likelihood of false-positive associations.

### 4.4 AMF enhanced microbial network complexity and ecological stability

Co-occurrence network analysis revealed that AMF-inoculated soils exhibited higher connectivity and modularity, representing more interactive and resilient microbial structures. Similar trends have been reported in AMF-inoculated wheat and tomato systems, where higher network complexity correlated with increased ecosystem stability and stress tolerance (Neu et al., 2021; He et al., 2025; Li et al., 2025). In contrast, the salt-stressed treatment showed fewer nodes and weaker interactions, indicative of a simplified and fragile network.

Mapping of csASVs within network modules demonstrated that AMF-related treatments were enriched in Actinobacteria, Firmicutes, and Proteobacteria, which are known to enhance stress resistance and nutrient cycling (Liu et al., 2024). These findings align with recent ecological models proposing that AMF strengthen interspecies cooperation, promoting functional cohesion and community resilience under stress (Shi et al., 2023).

Overall, this study demonstrates that AMF inoculation simultaneously benefits plant growth and reshapes the rhizosphere microbial network under salt stress. The enhanced microbial diversity, functional potential, and co-occurrence stability observed here highlight the ecological importance of AMF as key regulators of plant–microbe–soil interactions. By facilitating nutrient cycling, stabilizing microbial networks, and promoting host stress resilience, AMF symbiosis offers a sustainable strategy for improving crop productivity in saline–alkaline farmlands. Future studies integrating metagenomics and metabolomics are warranted to elucidate the molecular basis of AMF-mediated microbial assembly and to optimize their practical application in sustainable peanut cultivation.

Although formal null-model testing of modularity significance was not performed, the consistently higher connectivity, clustering coefficient, and modular organization observed in AMF-inoculated networks indicate enhanced robustness relative to salt-stressed soils. These comparative patterns suggest that AMF promotes structurally more resilient microbial networks under saline conditions. From an applied perspective, our findings suggest that AMF inoculation could be developed as a sustainable biofertilizer strategy to enhance crop productivity and stabilize soil microbial communities in saline–alkaline soils. Such AMF-based interventions may reduce reliance on chemical inputs while improving soil health and long-term agricultural resilience in salt-affected regions.

## 5 Conclusion

Overall, this study demonstrates that AMF play a key role in enhancing peanut performance and stabilizing rhizosphere microbial communities under salt stress. Beyond improving growth and yield, AMF-mediated restructuring of microbial networks and functional potential highlights their value as ecological regulators in saline soils. These findings underscore the potential of AMF-based strategies to support sustainable agriculture in salt-affected farmlands, offering a promising avenue for improving crop resilience, soil health, and long-term productivity under increasing soil salinization.

## Data Availability

The datasets presented in this study can be found in online repositories. The names of the repository/repositories and accession number(s) can be found in the article/Supplementary material.

## Supplementary material

SUPPLEMENTARY FIGURE S1 Root colonization of *arbuscular mycorrhizal fungi* (AMF) in peanut roots under different treatments. **(A)** Microscopic observation of AMF structures stained with trypan blue showing vesicles and hyphae in root cortical cells. **(B)** Percentage of AMF colonization in roots under four treatments. Data represent means ± SD (*n* = 3). ND indicates no detectable colonization. Asterisks denote significant differences compared with the control (*p* < 0.05). Stages: S = seedling stage; F = flowering stage; M = maturity stage. Treatments: C = control; A = AMF inoculation; S = salt stress; SA = AMF inoculation under salt stress.

SUPPLEMENTARY FIGURE S2 Canonical analysis of principal coordinates (CAP) illustrating bacterial community differentiation across treatments and growth stages. Each panel represents one treatment (C, A, S, SA), and symbols denote growth stages: seedling (S), flowering (F), and maturity (M). The percentages on axes indicate the variance explained by CAP1 and CAP2. Stages: S = seedling stage; F = flowering stage; M = maturity stage. Treatments: C = control; A = AMF inoculation; S = salt stress; SA = AMF inoculation under salt stress.

SUPPLEMENTARY FIGURE S3 Defining treatment sensitive bacteria in soil samples. Venn diagrams show the number of ASVs res ponding to treatment practices identified with indicator species analysis (purple) and by edgeR (cyan). ASVs identified by both methods were defined as cultivation sensitive ASVs (csASVs). Stages: S = seedling stage; F = flowering stage; M = maturity stage. Treatments: C = control; A = AMF inoculation; S = salt stress; SA = AMF inoculation under salt stress.

SUPPLEMENTARY FIGURE S4 Mean relative abundances (counts per millior1, CPM; log_2_ scale) of cultivation sensitive ASVs (as defined in Supplementary Figure S3, summarized at phylum level) across different treatment systems for soil bacteria. Stages: S = seedling stage; F = flowering stage; M = maturity stage. Treatments: C = control; A = AMF inoculation; S = salt stress; SA = AMF inoculation under salt stress.

SUPPLEMENTARY FIGURE S5 Mean relative abundances (counts per millioln, CPM; log_2_ scale) of cultivation sensitive ASVs identified by indicator species analysis and edgeR (see Supplementary Figure S3). ASVs are labeled with their family level taxonomy assignment, with the phylum level taxonomy assignment indicated by the colored bars. Stages: S = seedling stage; F = flowering stage; M = maturity stage. Treatments: C = control; A = AMF inoculation; S = salt stress; SA = AMF inoculation under salt stress.

SUPPLEMENTARY TABLE S1 An XLSX table reporting the indicator species and edgeR results and the assignments to cultivation sensitive ASVs and network modules.
