# Supplementary figures and images for: Arbuscular mycorrhizal fungi regulate the peanut rhizosphere microbiome to alleviate salinity stress and enhance yield
Source: Front Microbiol. 2026 Mar 18;16:1739241. doi: 10.3389/fmicb.2025.1739241 (PMC13042323; doi:10.3389/fmicb.2025.1739241)

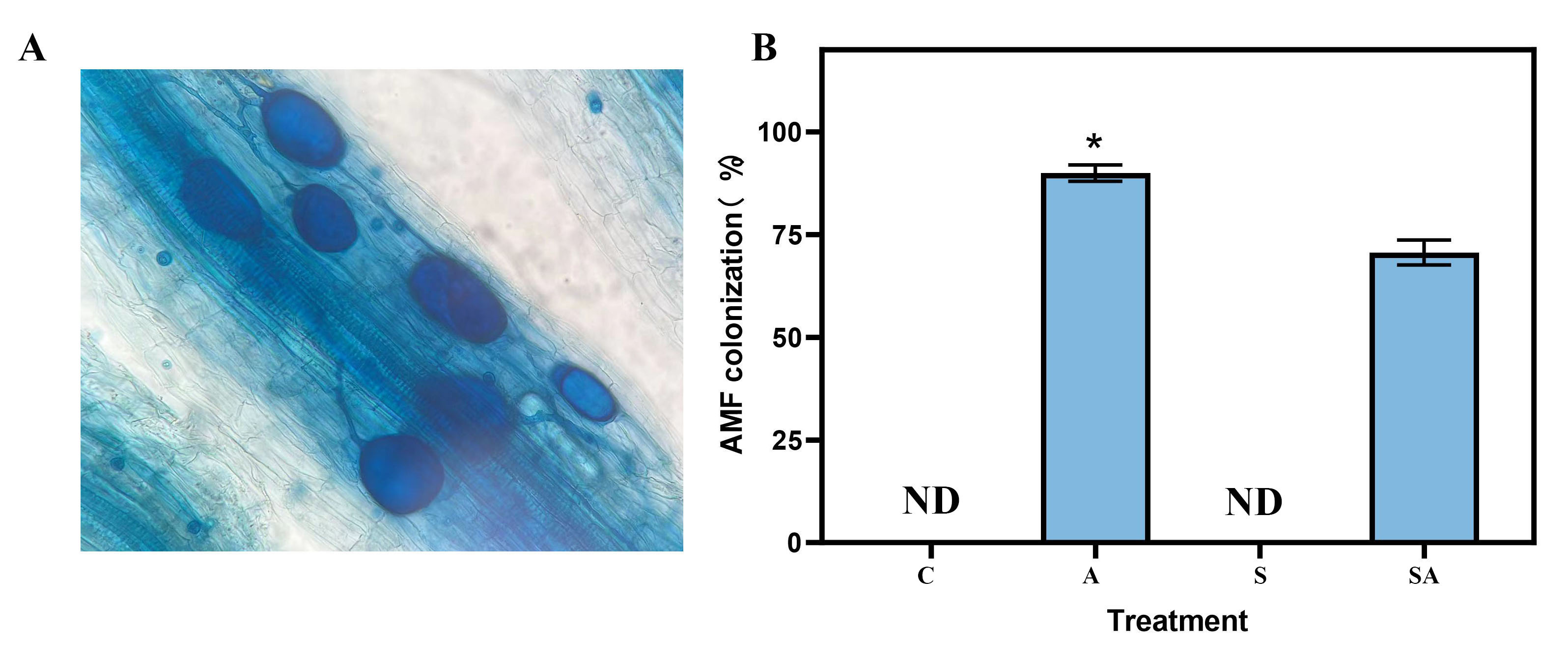

Supplement: SUPPLEMENTARY FIGURE S1 — Root colonization of arbuscular mycorrhizal fungi (AMF) in peanut roots under different treatments. (A) Microscopic observation of AMF structures stained with trypan blue showing vesicles and hyphae in root cortical cells. (B) Percentage of AMF colonization in roots under four treatments. Data represent means ± SD (n = 3). ND indicates no detectable colonization. Asterisks denote significant differences compared with the control (p < 0.05). Stages: S = seedling stage; F = flowering stage; M = maturity stage. Treatments: C = control; A = AMF inoculation; S = salt stress; SA = AMF inoculation under salt stress. [file Image_1.jpeg]

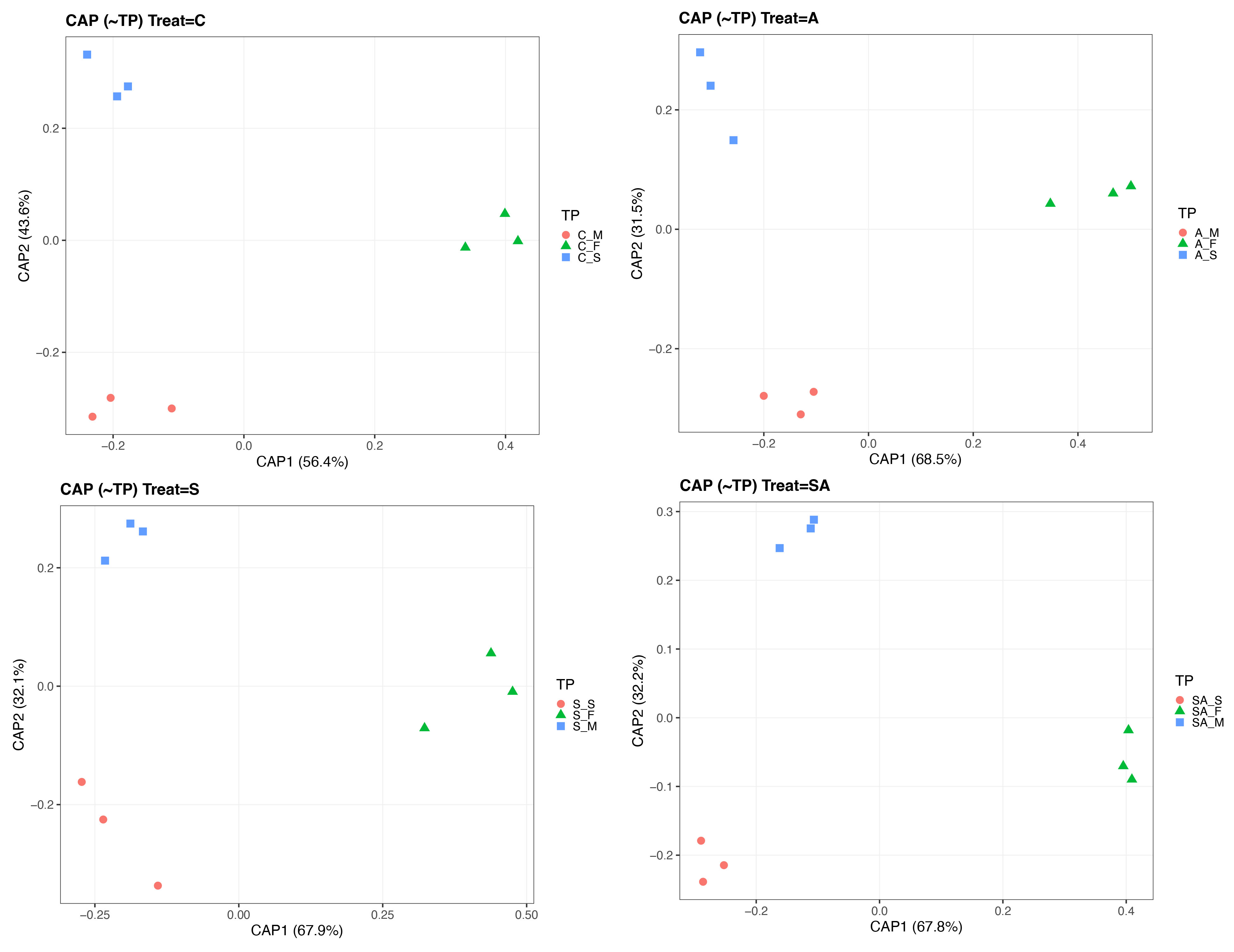

Supplement: SUPPLEMENTARY FIGURE S2 — Canonical analysis of principal coordinates (CAP) illustrating bacterial community differentiation across treatments and growth stages. Each panel represents one treatment (C, A, S, SA), and symbols denote growth stages: seedling (S), flowering (F), and maturity (M). The percentages on axes indicate the variance explained by CAP1 and CAP2. Stages: S = seedling stage; F = flowering stage; M = maturity stage. Treatments: C = control; A = AMF inoculation; S = salt stress; SA = AMF inoculation under salt stress. [file Image_2.jpeg]

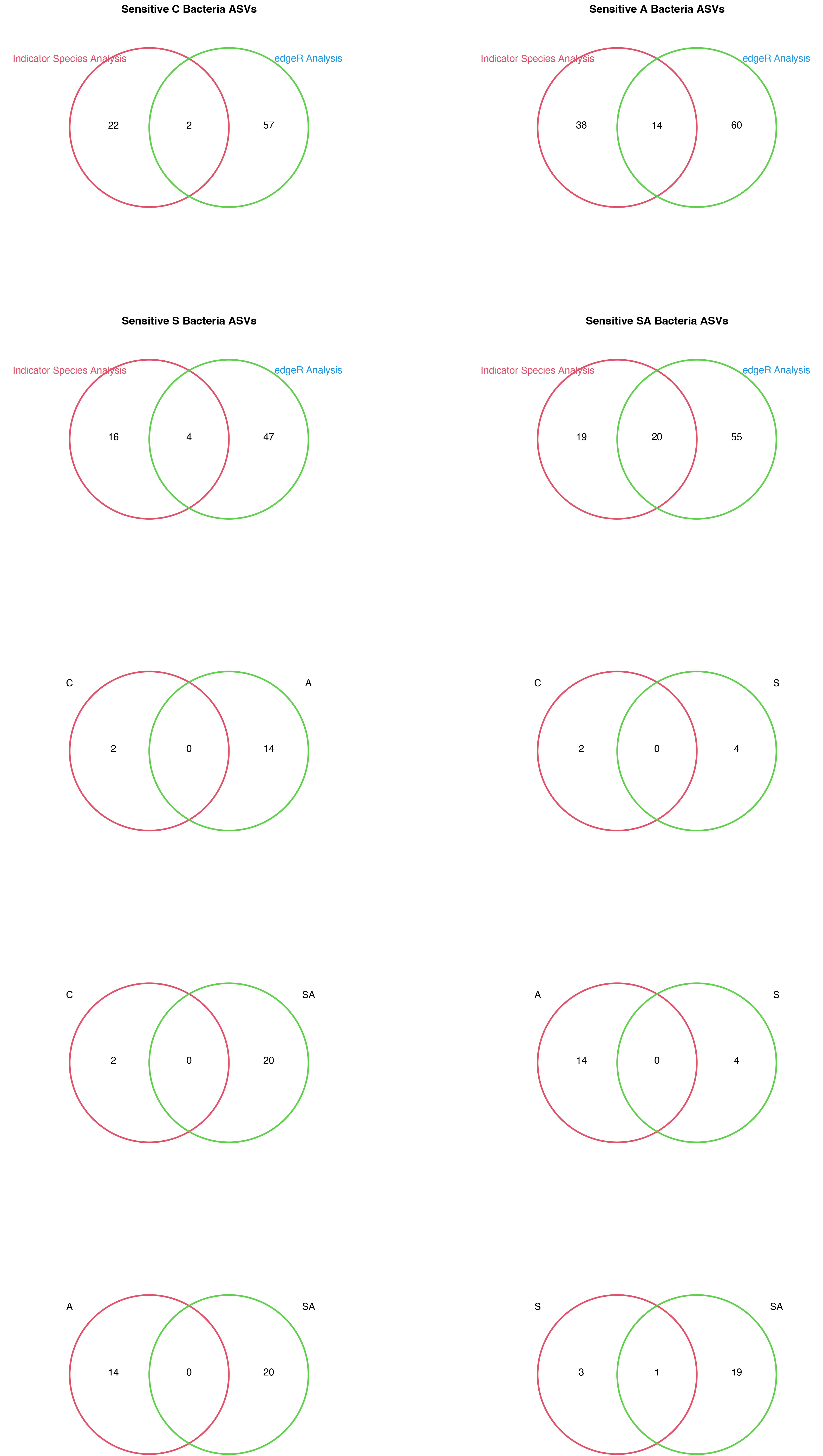

Supplement: SUPPLEMENTARY FIGURE S3 — Defining treatment sensitive bacteria in soil samples. Venn diagrams show the number of ASVs res ponding to treatment practices identified with indicator species analysis (purple) and by edgeR (cyan). ASVs identified by both methods were defined as cultivation sensitive ASVs (csASVs). Stages: S = seedling stage; F = flowering stage; M = maturity stage. Treatments: C = control; A = AMF inoculation; S = salt stress; SA = AMF inoculation under salt stress. [file Image_3.jpeg]

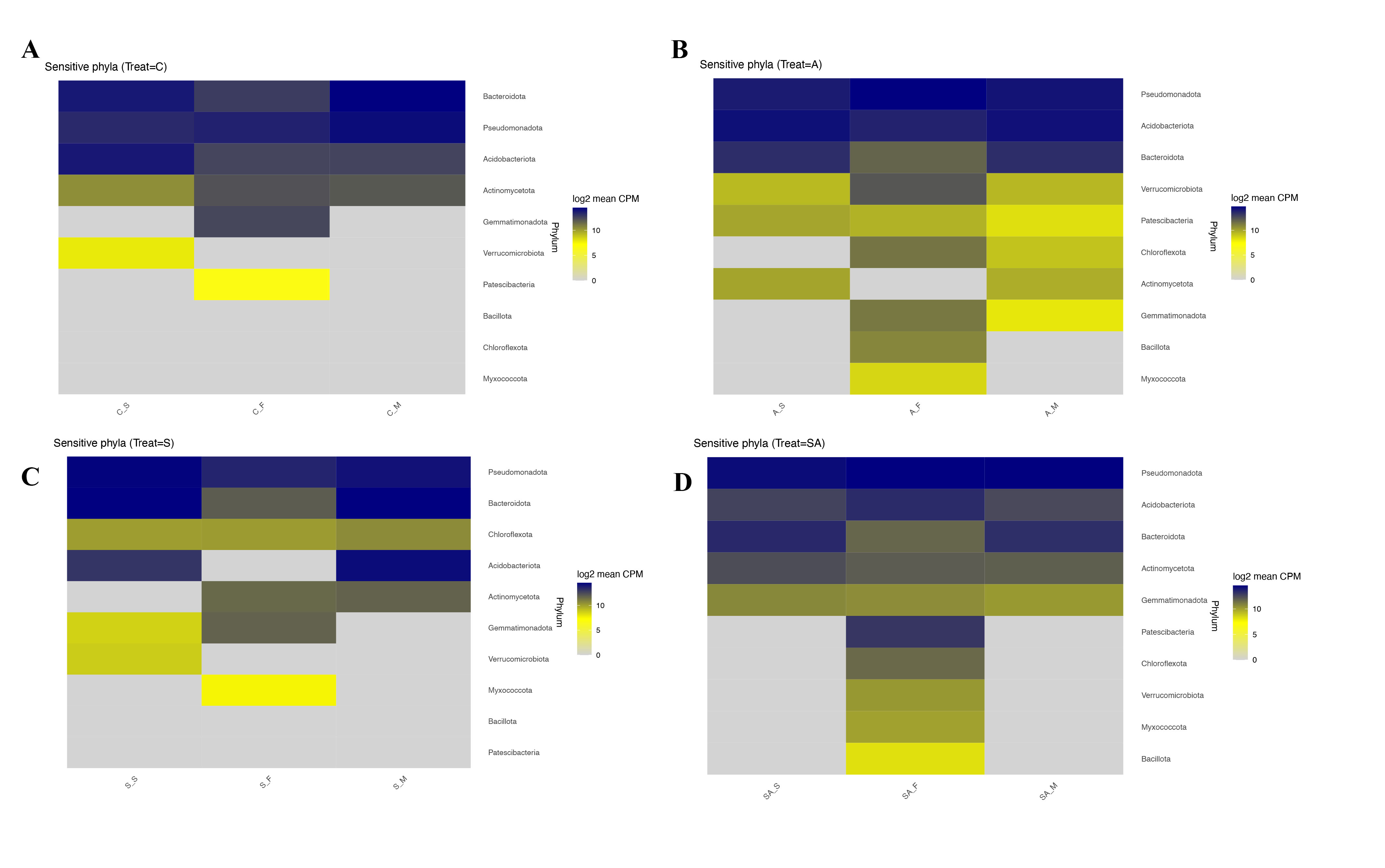

Supplement: SUPPLEMENTARY FIGURE S4 — Mean relative abundances (counts per millior1, CPM; log2 scale) of cultivation sensitive ASVs (as defined in Supplementary Figure S3, summarized at phylum level) across different treatment systems for soil bacteria. Stages: S = seedling stage; F = flowering stage; M = maturity stage. Treatments: C = control; A = AMF inoculation; S = salt stress; SA = AMF inoculation under salt stress. [file Image_4.jpeg]

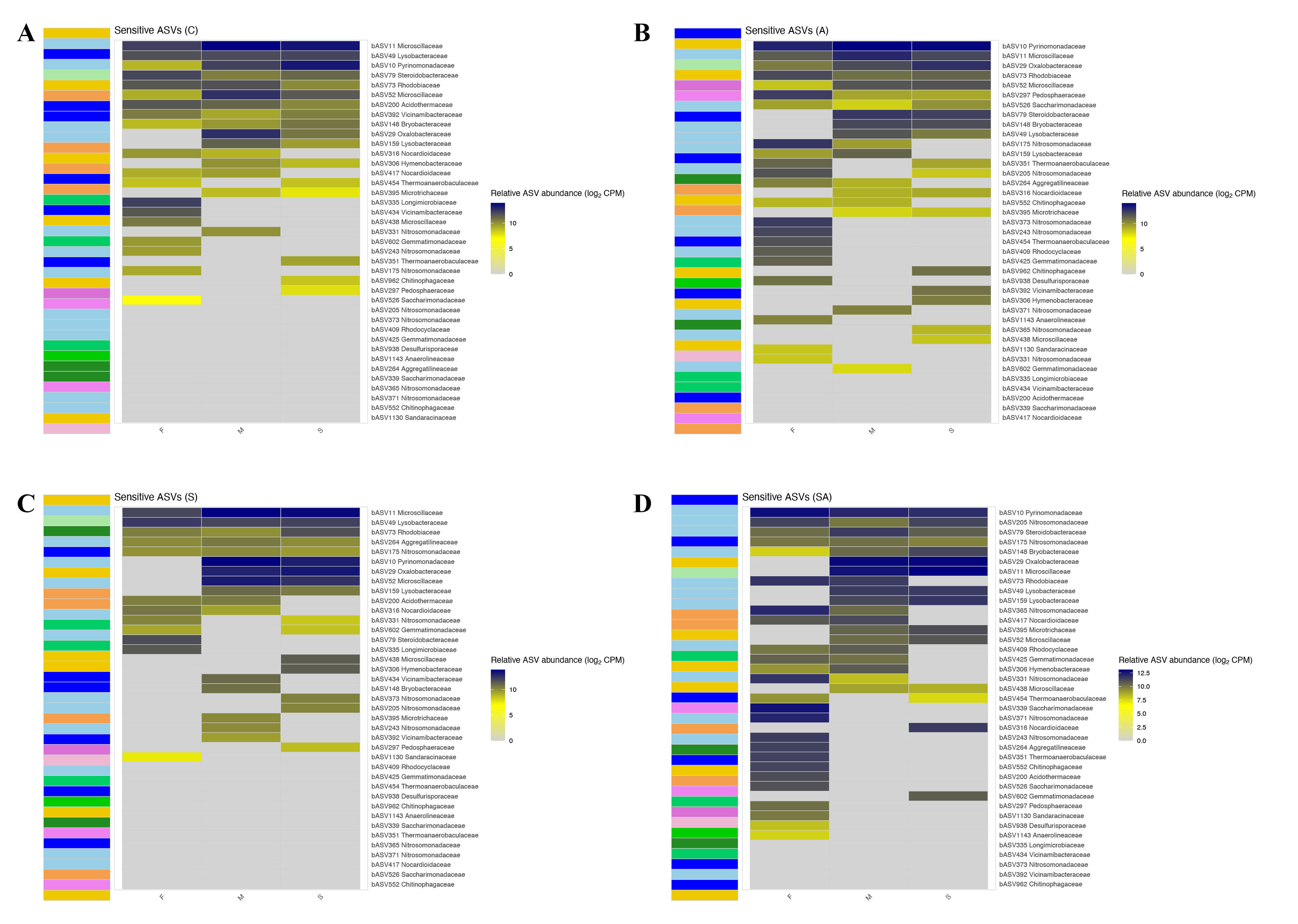

Supplement: SUPPLEMENTARY FIGURE S5 — Mean relative abundances (counts per millioln, CPM; log2 scale) of cultivation sensitive ASVs identified by indicator species analysis and edgeR (see Supplementary Figure S3). ASVs are labeled with their family level taxonomy assignment, with the phylum level taxonomy assignment indicated by the colored bars. Stages: S = seedling stage; F = flowering stage; M = maturity stage. Treatments: C = control; A = AMF inoculation; S = salt stress; SA = AMF inoculation under salt stress. [file Image_5.jpeg]
